# Facilitators and Barriers to Assistance Dog Puppy Raisers’ Engagement in Recommended Raising Practices

**DOI:** 10.3390/ani11051195

**Published:** 2021-04-21

**Authors:** Dac Mai, Tiffani Howell, Pree Benton, Virginia Lewis, Lynette Evans, Pauleen C. Bennett

**Affiliations:** 1 Anthrozoology Research Group, Department of Psychology and Counselling, School of Psychology and Public Health, La Trobe University, Flora Hill, VIC 3552, Australia; T.Howell@latrobe.edu.au (T.H.); Pauleen.Bennett@latrobe.edu.au (P.C.B.); 2 Centre for Service and Therapy Dogs Australia, Melbourne, VIC 3162, Australia; Pree.Benton@dogsforlife.com.au; 3 Australian Institute for Primary Care and Ageing, La Trobe University, Bundoora, VIC 3086, Australia; V.Lewis@latrobe.edu.au; 4 School of Psychological Science, La Trobe University, Bundoora, VIC 3086, Australia; L.Evans@latrobe.edu.au

**Keywords:** service dog, guide dog, puppy raising, puppy socialisation, dog training, organisational support, social support, puppy raiser, puppy walker, puppy foster carer

## Abstract

**Simple Summary:**

Raisers volunteer to raise and manage an assistance dog puppy for about a year and typically receive instructions for a wide range of puppy raising tasks from a host organisation. Those tasks vary among organisations, although the literature suggests that raisers should provide frequent socialisation and consistent training to their puppy, and engage in effective learning to improve their own practices. As those tasks are heavily embedded in the raisers’ daily lives, it is not easy to determine if any factors could affect their puppy raising. In this study, we interviewed eight puppy raisers monthly during their participation in an 11-month puppy raising program based at a university campus. Raisers thought that their puppies received more socialisation when they had more availability or someone else to share this responsibility with them, or when the puppies behaved well. Raisers could train their puppy more consistently when they had been prepared to deal with different scenarios occurring during their daily activities. While raisers found that some learning methods suited them better, they generally appreciated opportunities to learn, seek help from, and practise with other raisers. We hope these findings will inform development and evaluation of future programs aimed at improving practices and experiences of raisers.

**Abstract:**

Many assistance dog providers use volunteer raisers to manage each puppy’s learning and daily experiences, which partly determines the puppy’s behavioural development. Therefore, it is important that raisers engage in recommended practices. Three common recommendations from the literature include frequent socialisation and consistent training for the puppies, and effective training for the raisers. However, what facilitates or hinders raisers’ engagement in these practices remains unclear. To understand this, we interviewed eight raisers (three men and five women) every month during their year-long puppy raising program, and pseudo-randomly selected 16 from 48 interviews for data analysis. Thematic analyses revealed several facilitating and/or hindering factors corresponding to each of the three recommended practices. Frequent socialisation was influenced by the raisers’ availability, sharing of puppy raising responsibility with others, support from their workplace, and the puppy’s behaviours (e.g., soiling indoors, jumping). Consistent training was challenged by the presence of everyday distractors, accessibility to timely advice, perceived judgement from others, and the puppy’s undesirable behaviours. Effective learning was facilitated by having information available in raisers’ preferred learning modality, opportunities for peer-learning, and willingness to seek help. Future research should examine these factors quantitatively, which will enable more robust evaluation of programs aimed at supporting puppy raisers.

## 1. Introduction

The assistance dog industry provides certified dogs who are healthy and well-trained, to accompany and assist a human handler, certain aspects of whose daily functioning are affected by a disability, to live more independently [1,2]. The process of training and certifying assistance dogs normally begins with breeding and selecting puppies with suitable traits, then placing them with a volunteer who raises the puppy and assists the assistance dog provider by undertaking the many tasks required to produce well-rounded adult dogs [3,4,5,6]. As puppies need to be house-broken and socialised, a production model where puppies grow up in kennels is not appropriate for their welfare and learning objectives. Therefore, providers often rely on volunteer raisers to provide a safe home for the puppy’s first year of life, while the puppy begins early socialisation and training. At the end of this process, about half of the dogs do not become assistance dogs, mostly because their temperament and performance do not meet the industry’s strict criteria for skill competency and public safety [7,8,9]. Understanding why only some dogs succeed is key to optimising production.

It is well established that both nature and nurture drive the development of personality and behavioural patterns [10]. In a critical review, Mai et al. [11] argued that while research has focused on nature factors (i.e., selecting puppies with sound temperaments), very little is known about how management of a puppy’s learning and their experiences during their stay with the raiser affect success. During the puppy raising program, puppies navigate through their puppyhood, juvenile, and young adulthood stages [12,13,14]. Throughout this time, they are not socially mature, a neurological and developmental outcome which normally occurs when dogs are two to three years old [15,16,17,18,19]. The developmental process can be challenging and includes sensitive periods in which puppies are particularly fearful and susceptible to various stimuli [13,15,20,21,22]. This means that experiences during this time, and, in particular, the training approach used by those responsible for the puppy’s care, may be very influential. Literature is beginning to emerge which demonstrates better outcomes for companion dogs trained using primarily positive reinforcement relative to other approaches [22,23,24,25]. Without going into detail about the effectiveness of different dog training techniques, Mai et al. [11] asserted that, regardless of how puppies are bred or what policies and procedures are employed by the organisations that produce them, a critical link in understanding outcomes for individual puppies is knowing more about the practices actually engaged in, on a day-to-day basis, by those who raise them. At most, organisations can indirectly influence outcomes by affecting the practices engaged in by raisers, practices that are likely to also reflect many other influences. It is the raisers’ practices (including training and socialising practices) that are most likely to have direct influences on puppy behaviour, an assertion that was later supported by Mai et al. [26].

While raisers’ practices may be expected to have direct influences on puppy behaviour during critical developmental stages and beyond [26], only a small number of studies have focused on puppy raisers [3,4,5,6,26]. These typically did not specifically examine raisers’ practices but investigated puppy experiences. For instance, Chur-Hansen et al. [4] interviewed puppy raisers and subsequently raised concerns about the experiences of first-time raisers from one program. This study revealed several challenges, such as a lack of proper preparation, demanding workload, struggling with puppy training, experiencing negative emotions, and reduced motivation. Mai et al. [3] interviewed experienced raisers and staff from different organisations to explore factors thought to be associated with successful puppy raising. These helping factors included raisers’ personal attributes (e.g., competency and motivation), the availability of external supports (e.g., organisation’s technical instructions, emotional support from other raisers), and the nature of the puppy being raised. What raisers should do to help with the progress of their puppy and what factors could facilitate or hinder their engagement in those practices remains unknown.

Several reviews [22,27,28] and associated industry reports [29] concerning best practices in rearing and training of working dogs in other contexts (e.g., racing, herding, sledding) have highlighted common practices along with recommendations to enhance dog welfare and ensure better performance outcomes. These include careful management of the puppy’s learning and their exposure to stimuli likely to be present in their adult environment, and appropriate education, training and support for their trainers and handlers [22,27,28,29]. Although assistance dogs perform different roles than dogs working in these other contexts, the same recommendations for effective puppy raising practices are likely to be relevant.

Of critical importance is the process of socialisation, i.e., providing puppies with opportunities to familiarise themselves with and become desensitised to everyday living situations that they will likely encounter during their working life [13,22,30]. For livestock guarding dogs, this will include exposure to farm personnel and stock. For racing greyhounds, this will include exposure to the track, the lure, and other dogs. For assistance dogs expected to spend their adult lives working closely with humans, this might include appropriately supported exposure to public transport, shopping centres, crowded outdoor markets, and small children. Important also is training puppies to perform appropriate behaviours or life skills, e.g., walking on a leash, following commands for sit, down, and wait [27,28]. It is reasonable to suggest that many of these should be taught during the socialisation process, as the puppies will be required to perform specific learned behaviours in various environmental settings and in the presence of many distractions. 

A third critical practice to emerge from the working dog literature is for the puppy trainer or, in this current context, puppy raisers, to engage in ongoing education and training [27,29]. The effects of raisers’ acquisition of knowledge and skills on their puppies’ outcomes are not well documented except for a few tentative suggestions for improving raisers’ competency derived from qualitative research [3,4] and unpublished doctoral research [31]. While raisers may take time to gain experience and develop their competence before acquiring a puppy, engaging in professional development such as learning new knowledge and skills throughout the puppy raising process is an essential practice. Therefore, raisers’ engagement in effective ongoing learning appears to be instrumental to raising successful puppies [27,29].

In brief, there is currently no consensus on standards for best practices in puppy raising in the assistance dog literature. However, recommendations from working dog research provide a potential framework for guiding research in this area. Based on the available literature, we contend that puppy raisers should be encouraged to engage in frequent socialisation and consistent training for their puppy, and effective continuous learning for themselves. To identify facilitators and barriers to engagement in these recommended puppy raising practices, we conducted interviews with staff and students who were participating as puppy raisers in an assistance dog raising program conducted within a university community. We had no control over the program the raisers were participating in and did not observe it in any detail. Therefore, we are unable to describe the level of advice provided or even the style of training used. Our intent was not to evaluate this program or to compare it with others, but to identify facilitators and barriers to practice that may then act as evaluative criteria for future program evaluation research.

## 2. Materials and Methods

### 2.1. Participants

A total of eight (3 men; 5 women) La Trobe University (LTU) community members participated in the current study. There were five students and three full-time staff whose ages ranged from 20 to 64 years old. All had volunteered in a campus-based puppy raising program run by an independent assistance dog provider. The staff held various non-academic positions, while the student participants were enrolled in either undergraduate or postgraduate courses. At the time of recruitment, the length of their affiliation with LTU was between six months and 10 years, with most around three years. The inclusion criteria for the current study included being 18 years or older and students or staff at the LTU campus in Bendigo, a city which is located in regional Victoria, Australia, with a population of around 150,000 as of 2016 [32]. There were no predetermined exclusion criteria in our study, although the decision to enrol an interested applicant into the puppy raising program remained with the program providers, based on their assessment of whether the participant could provide a safe home conducive to socialisation and training. While some participants had prior experiences with their family dogs and puppies, none had raised an assistance dog puppy before.

### 2.2. Materials

The interviews followed a semi-structured schedule (see Table S1) which focused on various aspects of the participant’s engagement with the program. The schedule was developed by experienced members of our research group in consultation with the organisation conducting the puppy raising program, and it was piloted on other members of the research group with puppy-raising experience. This process resulted in a schedule that was less prescriptive than initially planned. During their puppy raising, we asked the raisers to share their recent experiences in the puppy raiser role as well as feedback about the program’s design and operation. For these progress interviews, we started the conversations with the simple request of *“Tell me about your experience with puppy raising during the last month”*. We then sought further elaboration on the raisers’ experiences and identification of any issues that affected their experiences as puppy raisers or the puppy’s training progress. Since this was a longitudinal study, we also asked the raisers in subsequent interviews if there had been any changes in relation to the experiences that they previously reported.

### 2.3. Procedure

The puppy raising program and associated study were advertised to LTU community members via internal email distribution and official media and communication channels. Interested staff and students received an information package. Upon receipt of a signed consent form, they were invited to an initial interview about their motivations, expectations, and perceived suitability. These individual factors are program-independent and were already investigated in Chur-Hansen et al. [4] and Mai et al. [3]. Therefore, data from these initial interviews were not included in the current study as they were beyond the scope of this study. After the interview, the applicant’s contact details were forwarded to the assistance dog program provider for a suitability assessment, which was conducted as per their existing procedure. Participants who were deemed suitable for puppy raising by the provider received their puppy between February and April 2019. All puppies were selected by the assistance dog provider for inclusion in the program, and all were female (six Labrador Retrievers, two Lagotto Romagnoli). Each participant raised one assistance dog puppy at a time, for approximately one year.

The experienced program provider was responsible for all operations of the puppy raising program, such as arranging for health checks and veterinary care, describing training protocols and meeting costs associated with raising and training the puppies. This organisation provided necessary equipment to ensure the raisers could meet the puppy’s needs. Group training sessions ran weekly and took place on campus and in local shopping centres. During those sessions, trainers also discussed any concerns the raisers had in their puppy raising. Trainers were also available to provide support by phone as required.

Participants were interviewed at the end of their first week in the program, except for one participant, Adrian (participant’s pseudonym), who was unavailable until the end of the second week (see Table 1). Progress interviews took place every month, either in person or via teleconference, until each participant relinquished their puppy back to the organisation for advanced training. With the participants’ consent, de-identified information from the interviews was sometimes used to suggest program improvements to the provider. It was deemed ethically irresponsible not to share this information, given that it could affect outcomes for the puppies in the program or their eventual handler with a disability. Part of the rationale for raising puppies in a university environment was to enlist raisers in working directly with the provider to improve the operations of the program, either directly or indirectly via de-identified feedback provided via the research team.

During the program, a total of 48 interviews were conducted all by the first author (D.M.). These were audio-recorded, with lengths ranging from 11 to 63 min and an average of 28 min. With recurring cross-sectional data collection (i.e., at different time points), the current study resembles a longitudinal qualitative study by its design though not by its theoretical approach [33]. Thomson et al. [33] argue that a longitudinal study by default should consider both the emergence of themes at each time point (synchrony) and their changes through time (diachrony). Instead, the current study’s aim related more closely to that of a cross-sectional (i.e., identifying emerging themes) than a longitudinal study (i.e., focus on changes in emerging themes through time). For this reason, data were analysed for all participants at the transition stage, but we then randomly selected one progress interview per participant (see Table 1) for analysis. This method enabled us to capture their collective experiences at various stages (i.e., synchronically) from four weeks to 40 weeks in the program, but without going through all data collected each month, which would reflect a diachronic approach and was beyond the scope of the project. Recordings of the 16 selected interviews were transcribed and analysed using NVivo 12 [34].

### 2.4. Data Analysis

Data were analysed in two stages. The thematic analysis took place in Stage One, which adopted an inductive approach that allowed themes to emerge from the data [35]. During this stage, the first author thematically coded the data as per Braun and Clarke [36] separately for the transition and the progress interviews. In Stage Two, those themes were discussed among four authors (D.M, T.H., P.B., and P.C.B.) in several meetings to retain the themes that were relevant to the three recommended practices derived from the existing literature, namely providing puppies with sufficient socialisation and consistent training, and raisers’ adoption of effective learning strategies. This sequence allowed for an in-depth exploration of the data (Stage One), which is generally appraised as a strength of qualitative methodology [35], while ensuring the findings were specific to the research questions (Stage Two). The second stage reflects a theory-driven approach to analysing qualitative data in program implementation research, which highlights the advantages of applying categorisations of factors and their relationships from established theories and frameworks [37,38,39,40]. In the current study, we based our three main categorisations on literature reviews on recommended practices in the working dog industry [22,27,29]. 

For each emerging theme, direct quotes are provided to give voice to the raisers and to characterise the influences that those factors had on the raisers’ engagement in the corresponding recommendations. The quotes were sent back to each participant for member checking. Commonly spoken filler words such as “uh” and “erm”, were removed from the quotes. Where appropriate, to aid readability, square brackets with the ellipsis (i.e., […]) indicate an omission of irrelevant responses. Square brackets may also contain a word or a phrase to replace identifiable details or grammatical errors. Parentheses provide explanations or essential contexts for the quotes.

## 3. Results

This section describes factors affecting raisers’ engagement in practices recommended for their puppy raising, namely frequent socialisation, consistent training, and effective learning.

### 3.1. Frequent Socialisation

In the current study, raisers took their puppy with them to work or study activities at the university campus. Those activities took place in classrooms, lecture theatres, offices, meeting rooms and sports fields, and in the university cafeteria, library and student lounge. Some student participants lived on campus, so their puppy was exposed to the campus student residences. Participants also travelled with their puppy to public places outside of the campus, such as shopping centres, restaurants, and on public transport. To ensure their puppy could meet with people of different ages and appearances, some puppy raisers proactively contacted and arranged puppy events at local primary schools and attended festivals on campus, while other raisers received various visitors to their office as part of their regular operations. Overall, puppies accompanied their raiser to places for reasons ranging from personal to professional. The frequency of those opportunities varied amongst raisers and was dependent on individual factors, as described below.

#### 3.1.1. Puppy Behaviour

Socialising the puppies occurred either as planned trips or during spontaneous travel, when they accompanied their raiser to various places. In both cases, how the puppy generally behaved during those occasions determined the convenience of their presence and the raiser’s willingness to take the puppy with them on subsequent trips. Problematic issues included, but were not limited to, soiling indoors, barking, jumping, and pulling on the leash. When discussing raisers’ motivation, we referred to a ten-point scale, with ten being highly motivated to take the puppy out on spontaneous trips. Veronica responded:

*If she has not gone to the toilet, if she just refuses to go, two (out of 10 on motivation). Honestly, I have left her in the car [while I quickly run in] because I just [did not] want to deal with it. The last thing I want is for her defecating in a fresh food aisle. You do not want that near food. It is disgusting and it makes me look bad. It makes me look like I do not take the dog to the toilet often enough. I have had it three times and I’m over it. People look at me funny and I just hate it.*

*(Veronica)*

Although puppies’ toileting issues appeared to be inconvenient for raisers in most public places, because of official acknowledgement of the puppies’ presence on campus, some toileting accidents that happened on campus at the beginning of the program were able to be openly communicated with relevant personnel, and raisers were then reassured that such behaviours were expected. That helped improve campus accessibility. However, raisers’ experiences remained less positive when their puppy exhibited inappropriate toileting behaviours in other public places. For other raisers, puppies’ behavioural issues such as leash pulling posed a safety concern. As Kate explained:

*Her major problem is such high excitement levels around animals, especially dogs, that she lunges. She is so strong that if she lunges, she can pull me over or really injure me. It became almost a medical issue for me taking her out somewhere where there are dogs. She can really hurt me quite easily without realising it, because I do have back problems. If she goes, she could really easily mess something up.*

*(Kate)*

While some puppies were comfortable with handling when given to raisers, others took time to improve. As the puppies improved, the socialising experiences became less stressful for both the puppy and their raiser. On some occasions, the improvement reflected the joint efforts of the raisers and the program providers to address puppies’ undesirable behaviours in public places. On other occasions, improvements were probably a reflection that the puppies’ behaviour was changing as they matured. For instance, Jane shared the following improvement:

*She has matured a lot over the last few months and gotten a lot easier to handle. She is a lot calmer once I put the vest on her and she settles in a lot quicker. When I take her to lectures, it used to take her five minutes or so to settle down and just sit there but now she’ll come in, we’ll sit down and by the time I’ve got my books out, she’s lying down underneath the desk and just knows that that’s what she has to do.*

*(Jane)*

#### 3.1.2. Raisers’ Other Commitments

Raisers in the current study had work and study commitments that at times made the socialisation of their puppy inconvenient. For instance, Veronica described that:

*I have a tight schedule for some days. If I have a huge assignment due, […] then my motivation will be down to bring her to the shopping centre because it takes double the time to get anywhere with her. That is just the way it is, and yes, her toilet schedule. Just schedules. Toilet schedule and my schedule that is it. It is a time-sensitive thing and a toilet-sensitive thing. Other than that, then I would take her in [to the supermarket].*

*(Veronica)*

At times, the high workload from other commitments required raisers to evaluate the necessity of allowing the puppy to accompany them, which would provide their puppy with socialisation and also extend the planned trips; sensitive time management was an essential skill.

#### 3.1.3. Supplementary Supports

Raisers in the current study were either staff or full-time students with part-time employment. Although they could bring the puppy to the university in their office or classrooms, many required additional supports for their puppy’s socialisation. For this reason, some additional socialisers were recruited and trained by the assistance dog provider overseeing the program. These were students or staff who signed up to volunteer a few hours per week to take a puppy out and socialise her. Family or other household members were also called upon to provide additional support. During days when raisers were at work or studying, they might ask their socialisers to pick up the puppy for a walk and to give her some training. Edwin attributed some improvements they observed in the puppy to the support from the socialisers:

*She (the puppy) could sleep all day if she wanted to, but I think she wouldn’t get enough training (and socialisation). Now, the socialisers take her out and they train her. I can actually say that because she gets more and more training, she’s picking the new things up a lot quicker so it’s not just me.*

*(Edwin)*

Wesley appreciated the support from another household member with the morning walk: “One of my housemates gets up at 6:30 every day and walks [the puppy] as part of [their] fitness thing”. For raisers who had disapproving housemates and less engaged socialisers, their puppy raising was more challenging. Veronica explained how their experiences improved with engaging puppy socialisers:

*My two (socialisers) I’ve got right now are pretty good, so I’m happy at the moment. They don’t seem to be indicating dropping out, so I don’t have any issue. But before, I did have an issue. I was left with one socialiser and [that socialiser] wasn’t available when I needed, so I had to leave the dog at home occasionally. That’s not ideal because [the puppy] is meant to be in training the whole time.*

*(Veronica)*

#### 3.1.4. Workplace Support for the Puppy

Being able to integrate puppy socialisation with other commitments presented a convenient opportunity. Veronica explained how bringing their puppy to the university allowed them to fulfil their study commitments while allowing the puppy to experience various public settings on campus: *“On bad days we’d get one walk in, but I also took her to [the university], so she’d get a walk around [campus].”*

This integration of the puppy into different activities on campus was generally welcomed. As Adrian expressed: *“Everybody knows about her, and they love seeing her at meetings”*. Other raisers similarly reported this. For instance, Harriet’s puppy went with them to work daily and could enjoy interactions with students and other staff in their office:

*Sometimes she’ll sit on that mat and she’ll watch where I am, or she’ll sit under my feet under the desk. If [my colleague] is here, she’ll (the puppy) often sit with her [in the] morning, but keep an eye on where I am.*

*(Harriet)*

The campus had received approval from university management to establish dog-friendly facilities (e.g., dog drinking bowl, suitable places for toileting, and the Anthrozoology Research Group Dog Lab where the weekly training took place), and relevant protocols were developed to ensure welfare and safety of both the canine and human members of the university.

### 3.2. Consistency in Training

In addition to extensive socialisation, it is widely acknowledged that puppies benefit from appropriate training [27,41]. During the puppy raising program, puppies and their raisers received weekly group training, and raisers could access trainers via private social media groups or by telephone outside of training sessions, if needed. There was no restriction on how often raisers could contact trainers or situations to not reach out to the trainers. The extent to which raisers were able to implement what they learned in these sessions was, however, quite variable. Despite their efforts to adhere to recommended training protocols, raisers reported several factors that either facilitated or impeded their capacity to follow the instructions provided.

#### 3.2.1. Puppy Behaviours

Raisers’ tolerance of their puppy’s behaviours during training appeared to affect raisers’ adherence to training protocols. Instructions were put in place to help puppies learn appropriate manners and to discourage inappropriate behaviours. However, the process of achieving these results was unpleasant for some raisers, who tended to relax their training regime to ameliorate the inconvenience of dealing with challenging behaviours. Kate described this process:

*When she barks, she squeals, which is part of why it is so stressful, [and] I think is just like a biological thing of responding to babies when they scream. When she was high pitched, I’m like, “It’s so high anxiety for me,” so I let her out of the crate because I’m like, “I just can’t handle it.” I think that this probably hindered the crate training a little bit just because she stresses me out so much. I do not put her in there very often.*

*(Kate)*

#### 3.2.2. Preparedness for Unanticipated Distractors

Raisers planned training sessions for their puppy, during which they could anticipate potential issues and prepare for these accordingly. However, most training occurred in different settings during the raiser’s daily activities, such as when they were at work, shopping, or studying. Adrian described situations where they had to walk the puppy in the presence of distractors that were inevitable in their workplace:

*[The trainer] just said to start trying to get her (the puppy) to walk on the left, so I try to assert for her to move on the left, but it’s quite difficult because, when she’s at university in the corridor, she wants to be on the right because she wants to talk to the people who are coming down in the opposite direction. I would have to shorten the leash down to the point where it was a third the length, and effectively she was having to walk rigidly beside me, which [the trainer] also says isn’t ideal.*

*(Adrian)*

Some distractors might also be present at home, which was also a hindrance to raisers’ ability to provide consistent training. Veronica shared their difficulty when trying to keep the puppy calm at home:

*If someone is excited to see her, she will be excited to see them. If someone is not happy [or indifferent] to see her, she generally will not react. Unfortunately, in the case of the person I live with, [my puppy is] very excited to see them almost all the time (because they hype her up against my wishes), so that can be a problem.*

*(Veronica)*

These distractions, encountered at home and in the community, meant that even when raisers understood the requirement to carry out training consistently and in line with specific instructions, in some situations they were unable to do this. It was difficult for raisers to be fully prepared for all situations, and it was particularly difficult when they were engaged in other matters and the puppy was not their primary focus.

#### 3.2.3. Accessibility to Timely Advice

It appeared in this study that strict adherence to the organisations’ instructions depended on raisers’ having ready access to protocols that applied to specific situations. Although some protocols were made available, it was not practical that they could cover all possible situations. Therefore, raisers found it helpful when they were able to reach out to the organisation for advice regarding situations when they felt uncertain. For instance, Fiona stated: *“[I] double-check everything twice with [the organisation] to make sure because I do not want to do anything that was going to jeopardize her.”*

However, it was not always practical for the raisers to obtain timely advice from the organisation for incidents that required immediate responses. While the organisation typically had specific instructions for how to interact with the puppy, Jane described other improvised strategies she used in uncertain situations:

*It has been working pretty well if you distract her with a toy (as recommended) and then sometimes if she gets a little bit hyperactive, then the toy does not necessarily always work. I might take her for a walk then or something to try and burn some of her energy.*

*(Jane)*

The raisers’ self-reported reactions to those situations were mostly based on their best judgement and experience and were often appropriate. However, it is not ideal for program adherence if raisers regularly have to rely on their own judgement. Having access to advice is critical, as is training raisers sufficiently so that they can troubleshoot effectively. Puppy raiser training is discussed later in this paper.

In short, for raisers to consistently carry out the training instructions provided, it was necessary that they were aware of the relevant protocols or able to seek immediate advice from their program provider for different situations, such as managing puppies’ temperament or their safety in public places. However, there were many situations where it was not practical for raisers to obtain timely advice from the organisation or where it was inconvenient for them to apply a particular training method.

#### 3.2.4. Perceived Judgement of Training Techniques

Puppy training can be a controversial process which requires a careful balance between positive reinforcement of desired behaviours, coupled with effective and humane techniques for preventing or discouraging undesirable behaviours. Perceived negative judgements of training techniques made by others hindered some raisers’ confidence to perform these techniques in public places. It is important to note that members of the public do sometimes comment on specific puppy-raising situations in ways that are judgemental, even when the techniques being applied are benign. For example, one of the authors of this paper has been reprimanded for using a hand to gently guide a puppy into a sitting position while waiting at a street corner to cross a busy road, and has also been instructed by well-meaning members of the public not to use food rewards while training a puppy to sit quietly in a shopping centre as they may “spoil” the puppy. The point here is not to debate the value of different training practices, but to highlight that perceptions of public judgement can interfere with the raising process. Veronica shared their sentiment regarding how other people’s opinions about their training techniques might have had an impact on their confidence to perform the tasks in public places:

*If people do not train the dogs, they do not understand what’s going on (when we use certain techniques especially regarding lead training and barking distraction training, use a halti or have certain rules like not running off lead or not getting pats). They think it is [not acceptable], so we […] do (some techniques) indoors, away from people. Which we have been doing a bit, and she is getting the point. She is getting there (getting better with her training/behaviour). It is mostly with her meeting people and other dogs really. […] If someone [comes] up to me and [says], that’s [not acceptable], then hopefully, I [am] with [another puppy raiser] or any of the dog people who might help me speak up (justify training choices and why they are not harmful).*

*(Veronica)*

In planned training and socialisation sessions, raisers could join each other and provide support for technical and emotional issues. However, with daily encounters for which the raisers did not specifically plan, upholding consistent training for their puppy appeared to be challenging. 

### 3.3. Effective Learning

During the puppy raising program, raisers were required to learn appropriate strategies and skills to guide their puppy’s learning and to help them navigate multiple developmental stages. Promoting raisers’ engagement in effective learning required efforts from the program provider, both formally through the provision of instructions, and informally through the endorsement of peer-support. It also required commitment from the raisers, who needed to engage in help-seeking behaviours and active learning strategies. Several factors influenced how successful this process was.

#### 3.3.1. Raiser’s Learning Style

Raisers had different preferences regarding what and how they would like to learn from the organisation. In the beginning, raisers preferred to be provided with more information and knowledge, either via written instructional materials or in-person inquiries with the organisation’s staff. Then, further into the program, raisers’ preferences shifted to in-person instructions that they received during the weekly training sessions. Edwin described this change in their preferred mode of instruction: *“I’ve probably been following more of what [the trainer] says, but less on the paperwork.”*

A benefit of in-person instructions that Edwin mentioned included the opportunity to observe step-by-step demonstrations by trainers. Other favoured features of the training sessions included the scaffolded design of teaching skills, and trainers’ knowledge and expertise. Wesley commented on the weekly sessions:

*I think the instructions are straightforward and what [the trainer] demonstrates are immediate effective results that he can demonstrate. That’s a powerful learning tool from a learner’s perspective. He clearly understands the language of the dog, and they understand that from him. He delivers training progressively. Each week there is a new skill, but reiteration of the previous week’s skill, which is an effective learning strategy.*

*(Wesley)*

The provision of simple instructions helped some raisers address their puppy’s immediate issues, although other raisers expressed an interest in receiving more in-depth understanding of the training techniques. Kate recalled a question they had for the trainer early in the program:

*I think that [detailed explanations] were missing a little bit early on. I used to say all the time that if [the trainer] can look at the dog, see the problem, [he can] tell you what the solution is, but he doesn’t show his work. […] You just sort of do what you’re told because you were told to do it, which absolutely makes sense because they’re dealing with a lot of people who don’t necessarily know about the dogs, but it can really help.*

*(Kate)*

Overall, although written instructions were helpful mostly during the early stages of the program, where they provided information to meet the raisers’ frequently asked questions, raisers tended to differ in their preferred modes of learning and the level of detail they preferred as they and their puppies progressed in the program.

#### 3.3.2. Opportunity for Peer-Learning

Raisers often reported benefits of learning from and sharing experiences with other raisers between the weekly formal training sessions. Fiona distinguished the informal advice from other raisers from the formal weekly instructions provided by the organisation:

*The organisation is more instructional and then they can answer questions and stuff like that, but it’s only once a week. If things come up in the week, that’s when I feel like the other puppy raisers and socializers are helpful […]. If there’s something that I have trouble with, if I run into someone or if I have a question, I can either post it on Facebook (in a private group that was exclusively accessed by the raisers and socialisers in the current study) or send someone a message.*

*(Fiona)*

Raisers needed to practise the skills they learned in the weekly instruction sessions. Therefore, assisting one another in this process benefited the raisers’ learning and training for their puppies. Kate described some in-field training activities they had with the other raisers: *“A couple of weeks ago I was able to go do some recall training with Wesley and [Wesley’s puppy] because [they] needed some work on that. We went out and did some work together.”* In addition, when raisers encountered puppy behavioural issues, they often checked in with the other raisers before deciding whether to escalate the issues to the trainer. The availability of other raisers not only facilitated their learning by allowing for informal inquiries but also provided more opportunities to practice the skills they had learned during the training sessions.

#### 3.3.3. Help-Seeking Attitude

Not seeking help was a barrier to effective learning and skills acquisition. In the current study, raisers reported two reasons for not contacting the organisation despite knowing that it would have been helpful if they had done so. First, they might have already asked many questions before and so they were afraid of bothering the training staff. This hesitation was still relevant even when the organisation explicitly encouraged them to reach out if they had any concerns. Fiona maintained:

*I do think [the trainer] did emphasize that if there is a problem, it’s best to say there is a problem, as opposed to just trying to suffer it out, because sometimes the fix is just so quick and easy. I remember them saying that before, but for some reason, I still felt a little bit trepidatious about bothering them too much.*

*(Fiona)*

Raisers suggested that it would help to include in the *“instruction book they gave us [about the] do’s and don’ts in different situations so that we don’t have to always ask [and wait for a response]”* (Veronica). 

The second reason affecting raisers’ willingness to reach out for help related to how they perceived staff’s availability and responsiveness. Early in the program, Adrian shared that *“[the training staff] want us to contact them 24/7, but they take a long time to get back to you.” Therefore, their suggestion was to have more staff available to help answer raisers’* questions.

Despite the staff’s busy schedules, after the organisation was informed about this issue, there was an improvement in the organisation’s responsiveness to the raisers’ inquiries. Wesley reviewed the staff’s responsiveness and said that *“I feel as if they respond within a good amount of time. It’s in the day. I know he’s busy.”* Jane echoed this sentiment and described this improvement in more detail:

*If I send them a question in the WhatsApp group, they’ll get back to me pretty quickly. They’re really informative and really passionate as well, which helps. It shows how motivated they are and helps you be motivated and things like that.*

*(Jane)*

Encouraging help-seeking did not seem to be sufficient. For frequently encountered issues, raisers suggested that having an easily accessed online and/or printed knowledge base would allow them to explore the answer to their inquiries instantly. It also appeared that organisations should be available to help answer their raisers’ concerns, especially when they first start to raise an assistance dog puppy.

**Figure 1:**
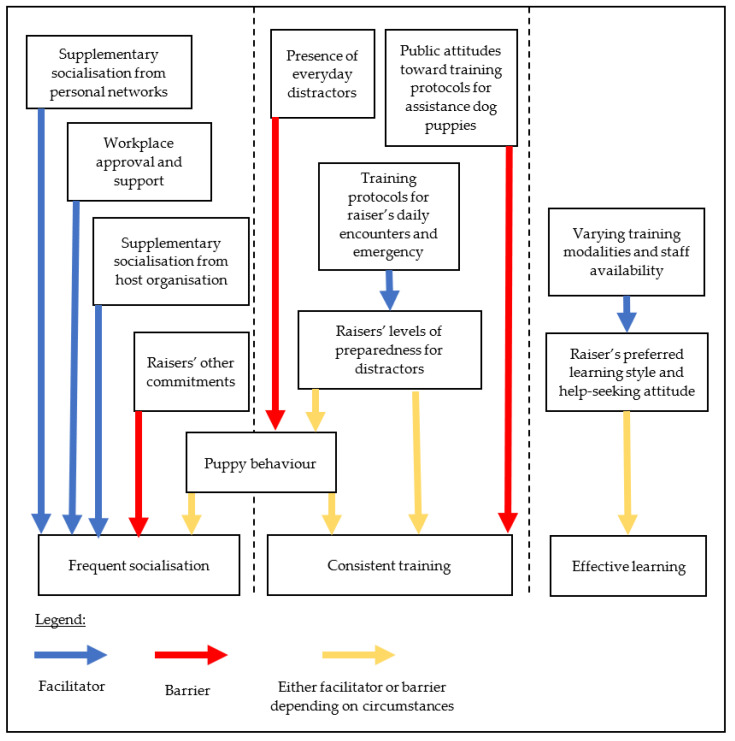
Multiple levels of facilitators and barriers to raiser’s engagement in recommended practices.

In summary, the current findings identified several factors that facilitated or hindered (or both, depending on the raisers’ circumstances) the puppy raisers’ engagement in frequent socialisation, consistent puppy training, and their ongoing effective learning. Those factors and their influences on the raisers’ practices are illustrated in Figure 1.

## 4. Discussion

The current study aimed to explore factors that facilitate or hinder volunteer puppy raisers’ engagement in recommended practices. The three recommended practices arising from the working dog literature [22,27,28,29] that pertain to assistance dog raisers include frequent socialisation and consistent training for the puppy, and engagement in effective ongoing learning for the raiser. The findings revealed four common themes that appeared to influence raisers’ provision of frequent socialisation opportunities for their puppy (see Figure 1). These included raisers’ other commitments, workplace acceptance and support, availability of additional supports, and the puppy’s behaviours. Factors influencing training consistency included raisers’ preparedness for everyday distractors, accessibility to timely advice, perceived public judgement of training techniques, and the puppy’s behaviour. For engagement in effective learning, factors such as suitability of instruction methods to the raisers’ learning preferences, availability of opportunities for peer learning, and their help-seeking attitude influenced success.

The current literature often refers to socialisation with a focus on the puppy, such as recommendations for how to manage their exposure [22]. In the current study, we explored socialisation from the perspective of the raisers and considered two behavioural aspects of raisers’ practice: creating socialisation opportunities for their puppy (i.e., frequency of socialisation) and consistently following instructions. The current study is the first, to our knowledge, to look at socialisation frequency as a specific aspect of raisers’ practice, though the factors identified in the current study reflect past findings in the assistance dog literature [3,4,5]. Raisers in Chur-Hansen et al.’s [4] study reported feeling overwhelmed by the workload associated with raising their puppy. With a more specific focus on the raisers’ practices, the current study found that a conflict between different priorities affected their puppy’s socialisation opportunities. 

Mai et al. [3] added to Chur-Hansen et al.’s [4] findings regarding the adverse effects of some puppies’ undesirable behaviours on the raisers’ experience. Mai et al. [3] found that raisers enjoyed raising an easy-going puppy, which made their raising experience easier and more positive. The current findings extended on this, in that some puppies’ disruptive behaviours made it less convenient and discouraged raisers from taking their puppy with them to many public places, which created a vicious circle that reduced the puppy’s opportunities to learn and improve their behaviours. It may seem straightforward to ask raisers to offer puppies ample public exposure opportunities; however, many everyday life factors, such as raisers’ availability and their puppy’s behaviour, could hinder a raiser’s initiation of those practices.

As Mai et al. [3] suggested, organisational support, which was reported as lacking from the program studied by Chur-Hansen et al. [4], could help raisers in many ways. In the current study, support from the program provider appeared to help raisers create socialisation opportunities for their puppy. Knowing that raisers in the current study were either full-time staff or students, the program provider involved their family members and housemates in the training sessions, and also recruited and provided training for other staff and students (socialisers) to assist the raisers. These supplementary supports were generally reported as helpful, allowing the raisers some breaks during the week or during their work and/or study. It also helped the raisers when the University offered support through acknowledging the program in their public advertisements and internal communications, and via provision of dog-friendly environments and safety features (e.g., the Anthrozoology Research Group Dog Lab, the automatic water bowl). 

For young puppies, training is largely inseparable from socialisation during outings [3,4]. Therefore, it is likely that these extra supporters also provided the puppies with some degree of training. The effectiveness of this additional training was not evaluated in the current study, but the supplementary socialisation provided in this program clearly increased the total number of hours spent socialising each puppy. Understanding its effectiveness requires further information about the competency and consistency of the supporters, which was not available. We also acknowledge that accessing extra personnel to perform the role of “socialiser” may not be possible in all contexts. Participating in a campus-based puppy raising program enables a large number of people to cooperate in the training process in a concentrated geographical area. 

Masinter [42] noticed a growing trend in raising assistance dogs amongst university or college students in the United States. While organisations and educational institutions must accommodate accredited service or assistance dogs, Masinter [42] argues that whether to offer access rights to assistance dog puppies, who are handled by puppy raisers, on their premises remains at their discretion as the puppies are not in formal training or supervised by certified dog trainers. Such decisions about public access rights of assistance dog puppies (who are being house-broken and socialised by puppy raisers) versus fully certified assistance dogs or assistance dog candidates being trained by qualified trainers need to be made in consultation with local authorities. We contend that colleges and universities offer environments that can be particularly well suited for assistance-dog puppy raising, particularly when it is possible to augment these programs with the additional support identified in the current study. This allowed raisers to leave their puppy with trained socialisers when they were at work or study, and also included provision of additional facilities for group training events. Although there have been previous reports of puppies being raised by university staff and students [43] and inmates [44], the focus was mostly on the effects of participation on the raisers. Future research should also evaluate the effectiveness of different puppy raising models in terms of cost and feasibility of program operation, and the outcomes for both the raisers and their puppy.

Regarding raisers’ training consistency, the current findings extend beyond the existing literature, which mostly concerns the welfare and effectiveness of various training methods and techniques [25,45,46]. One common feature in the dog training literature is the importance of consistency in training on behavioural learning in animals generally [45], and dogs specifically [47]. In puppy raising programs, raisers should closely and consistently follow instructions from their program provider. 

Strict and consistent adherence to program protocols may not be practical in some situations. The themes emerging from the current findings suggest several barriers to this process, which range from the raisers’ everyday encounters, through to perceived judgements from the public regarding particular training techniques, to the puppy’s own behaviour. The current findings reveal a more practical perspective in which spontaneous distractors and the occurrence of complex situations reported by raisers present real-life barriers that research and industry should consider in their recommendations and during development of training protocols. Implementing recommendations for best practices in training and socialisation may be possible in some circumstances. However, because daily life can be unstructured and unpredictable, collective efforts from the industry are required to further understand the myriad difficulties and challenges that arise in raisers’ lives, and how these might affect program adherence and subsequent dog behaviour outcomes. 

High demand for assistance dogs is a justification for recruiting inexperienced puppy raisers and allowing them to gain knowledge and skills as they raise their first puppy. Ideally, raisers will incorporate their experiences and knowledge from raising their first puppy into raising subsequent ones. However, it is not realistic to assume this is always the case, as struggling and demotivated raisers may not go on to raise another puppy [4,5]. It is not ethical to involve volunteers in a challenging role without considering their suitability and providing adequate support and training during their participation. Assistance Dog International [48] requires its member organisations to provide support and a training program to their volunteer raisers. Regardless of support, it is still necessary that the novice raisers meet some basic requirements. In acknowledging the challenges of raising puppies, some guide dog organisations require interested volunteer raisers to be assessed as physically and psychologically fit to the role [49]. The current findings resonate with those requirements, particularly when the raisers had to handle a large dog with a high level of energy and/or disruptive behaviours. In determining the nature of organisational support and approach to volunteer recruitment, the assistance dog industry could benefit from findings and frameworks in industrial–organisational (IO) psychology [50]. IO psychology focuses on how personnel recruitment, task design, and training would improve employees and volunteers’ job satisfaction and performance in not-for-profit sectors [50,51,52,53]. Mai et al. [11] have argued for a central role of puppy raisers in achieving better outcomes of the puppy raising process, which supports a potential application of IO psychology frameworks in assistance dog puppy raising research. 

Relevant also to first-time puppy raisers is the learning component of their puppy raising practice. As it can be demanding for raisers to learn new skills as they raise their puppy, it helps when they adopt effective learning strategies. Past research found that outcomes of puppy raising for first-time raisers were less favourable than for their experienced counterparts [54,55]. Mai et al. [3] suggested that, although more experience could enable raisers to increase their competency, organisations could accelerate this process by providing raisers with not only training and education but also with opportunities for supervised practice of relevant skills.

The current findings add to this suggestion by confirming that such opportunities could be provided through peer learning activities. Raisers are not professional dog trainers, which means that their advice for each other was informal and might not necessarily be as effective for certain issues as advice from the trainers. However, the raisers in our study reported several benefits of seeking help from other puppy raisers, and some preferred this over more formal advice. Peer-learning is not a new concept and benefits are manifold—it is cost-effective for the organisation, more accessible for the learners, and enables both learners and helpers to consolidate and strengthen their knowledge and skills [56,57]. If appropriately designed and managed, an official peer learning program also appears to address the other two barriers identified in the current study, i.e., instruction methods that do not suit raisers’ preferences, and raisers’ hesitation to engage in help-seeking. These barriers to raisers’ learning were also identified in Mai et al. [3] as factors affecting raisers’ experiences generally. While it may be logistically challenging to vary instruction methods to meet each raiser’s individual preferences, an organisation may provide advice and protocols for peer-learning activities that can then take different forms. They can also arrange socialisation and training sessions where raisers can interact and, as Mai et al. [3] suggested, set up an online discussion forum with experienced raisers or staff to act as mediators, ensuring the accuracy of any advice offered by peers and directing questions to trainers when necessary.

### Limitations

Different organisations have different program designs, and their volunteer raisers may have different work arrangements and life experiences than the raisers in the current study. The current raisers studied or worked at the same university campus and participated in the same puppy raising program. Therefore, the current findings may have limited applicability elsewhere. We also focused on exploring the raisers’ practices and did not collect data on which puppies were successful in advancing to the next stage of training. Furthermore, the opportunity to provide de-identified feedback through, and frequent contact with, the authors in this study could be extraneous factors that contributed to the raisers’ positive experiences and practices. Although the participatory action feature of this study is appropriate in applied social research [58] to protect the raisers from known challenges associated with raising an assistance dog puppy [4], more definitive and objective investigations of facilitating and hindering factors on raisers’ practices are required to address the limitations of this study.

## 5. Conclusions

Research on raising and training assistance dogs has focused extensively on puppy selection, with little attention being paid to another important factor: management of the puppy’s behavioural development by volunteer puppy raisers [11]. The current research aimed to identify factors that facilitate or hinder puppy raisers’ performance of effective practices, which has been a significant gap in the past literature. Three recommended practices emerged from reviewing the working dog literature, a broader category which covers assistance dogs. These were: providing frequent socialisation opportunities, adhering to consistent training protocols, and adopting effective learning strategies [22,27,28,29]. 

The findings of the current study revealed that factors hindering raisers’ provision of frequent socialisation for their puppy included having other commitments and the puppy’s disruptive behaviours. On the other hand, two factors facilitated this practice: receiving additional supports from other family members, friends, or socialisers assigned by the program provider, and approval and support from their workplace. Although raisers generally attempted to follow the program’s protocols, they reported several barriers. These included the presence of unpredictable and unavoidable distractors in their everyday situations, access to timely advice, perceived negative judgement from the public about their training techniques, and their tolerance of the puppy’s behavioural issues. To facilitate raisers’ effective learning, raisers suggested that comprehensive instructions and information should be provided in advance and made available in various modes to suit their preferences. Raisers also reported that having opportunities to learn from and practice with other raisers was helpful. In contrast, they were hesitant to approach trainers frequently for commonly encountered matters, most often out of concern for repeatedly bothering the trainers, despite these interactions being welcomed by the trainers. In understanding these emergent factors, the assistance dog industry could extend the use of their existing recommendations and puppy raising tasks, which are generally effective when they are performed in controlled settings and/or by experienced trainers and raisers, to be implemented reliably across all puppy raisers, particularly the less experienced ones. It is recommended that organisations recruit raisers who can meet the physical and behavioural demands of their puppy and pay sufficient attention to quality assurance of their organisational communication, peer-learning and mentoring programs.

## Figures and Tables

**Table 1:** Length in the program at the time of interviews included in data analysis.

Raisers’ Pseudonym	Weeks Since Arrival of Puppy	
	Transition Interview	Progress Interview
Adrian	2	4
Edwin	1	5
Wesley	1	16
Fiona	1	20
Jane	1	21
Veronica	1	26
Harriet	1	35
Kate	1	40

## Data Availability

The data are not publicly available due to privacy and ethical reasons.

## Supplementary Materials

The following are available online at https://www.mdpi.com/article/10.3390/ani11051195/s1, Table S1: Semi-structured interview schedules for puppy raisers.
