# Supplementary material for: Facilitators and Barriers to Assistance Dog Puppy Raisers’ Engagement in Recommended Raising Practices
Source: Animals (Basel). 2021 Apr 21;11(5):1195. doi: 10.3390/ani11051195 (PMC8143300; doi:10.3390/ani11051195)
Supplement: Supplementary file 1 [file animals-11-01195-s001.zip › animals-1188429-supplementary.pdf]

Supplementary Material

Facilitators and Barriers to Assistance Dog Puppy Raisers' Engagement in Recommended Raising Practices

Dac Mai [1,*], Tiffani Howell [1], Pree Benton [2], Virginia Lewis [3], Lynette Evans [4] and Pauleen C. Bennett [1]

[1] Anthrozoology Research Group, Department of Psychology and Counselling, School of Psychology and Public Health, La Trobe University, Flora Hill, VIC 3552, Australia; T.Howell@latrobe.edu.au (T.H.); Pauleen.Bennett@latrobe.edu.au (P.C.B.)
[2] Centre for Service and Therapy Dogs Australia, Melbourne, VIC 3162, Australia; Pree.Benton@dogsforlife.com.au
[3] Australian Institute for Primary Care and Ageing, La Trobe University, Bundoora, VIC 3086, Australia; V.Lewis@latrobe.edu.au
[4] School of Psychological Science, La Trobe University, Bundoora 3086, Victoria, Australia; L.Evans@latrobe.edu.au
* Correspondence: Jimmy.Mai@latrobe.edu.au; Tel.: +61-035-444-7317

Citation: Mai, D.; Howell, T.; Benton, P.; Lewis, V.; Evans, L.; Bennett, P.C. Facilitators and Barriers to Assistance Dog Puppy Raisers' Engagement in Recommended Raising Practices. *Animals* **2021**, *11*, x. https://doi.org/10.3390/xxxxx

Academic Editor: Kate Hill, Naomi Cogger and Mia Cobb

Received: 1 April 2021
Accepted: 20 April 2021
Published: date

Publisher's Note: MDPI stays neutral with regard to jurisdictional claims in published maps and institutional affiliations.

Copyright: © 2021 by the authors. Submitted for possible open access publication under the terms and conditions of the Creative Commons Attribution (CC BY) license (http://creativecommons.org/licenses/by/4.0/).

Table S1. Semi-structured interview schedules for puppy raisers.

**Opening Question**	
Tell me about your experience with the puppy raising during the last month.	
**Prompts**	
At home	Joy, difficulties associated with having the puppy at home; changes to daily routines/activities.
At work	In your office, in the classroom; attitudes towards others' reactions towards the puppy's present; changes to workplace dynamic and/or personal productivity.
Organisation	Attitudes towards levels of support received; feedback on program design.
Personal development	What have you learned about raising an assistance dog puppy?<br>Whether you learned that from our personnel or outside/online sources

Note: We will be focusing on the participants' experience. Not reporting on the puppy progress, but how the progress relates to their experience during the week. Duration: expected to be approximately 15 min.
